# Integration of Transcriptome and Metabolome Provides New Insights to Flavonoids Biosynthesis in *Dendrobium huoshanense*

**DOI:** 10.3389/fpls.2022.850090

**Published:** 2022-03-14

**Authors:** Yingdan Yuan, Jiajia Zuo, Hanyue Zhang, Runze Li, Maoyun Yu, Sian Liu

**Affiliations:** ^1^College of Horticulture and Plant Protection, Yangzhou University, Yangzhou, China; ^2^Anhui Tongjisheng Biotechnology Co., Ltd, Lu’an, China

**Keywords:** transcriptome, metabolome, medicinal plants, active ingredient, growth year

## Abstract

*Dendrobium huoshanense* is both a traditional herbal medicine and a plant of high ornamental and medicinal value. We used transcriptomics and metabolomics to investigate the effects of growth year on the secondary metabolites of *D. huoshanense* stems obtained from four different years of cultivation. In this study, a total of 428 differentially accumulated metabolites (DAMs) and 1802 differentially expressed genes (DEGs) were identified. The KEGG enrichment analysis of DEGs and DAMs revealed significant differences in “Flavonoid biosynthesis”, “Phenylpropanoid biosynthesis” and “Flavone and flavonol biosynthesis”. We summarize the biosynthesis pathway of flavonoids in *D. huoshanense*, providing new insights into the biosynthesis and regulation mechanisms of flavonoids in *D. huoshanense*. Additionally, we identified two candidate genes, *FLS* (*LOC110107557*) and *F3’H* (*LOC110095936*), which are highly involved in flavonoid biosynthesis pathway, by WGCNA analysis. The aim of this study is to investigate the effects of growth year on secondarily metabolites in the plant and provide a theoretical basis for determining a reasonable harvesting period for *D. huoshanense*.

## Introduction

*Dendrobium* is the second largest genus in the family Orchidaceae, with primarily tropical and subtropical distribution in Asia and Oceania (Jin et al., 2013; Xiang et al., 2013). *Dendrobium huoshanense* is a perennial herb belonging to the genus *Dendrobium* in the family Orchidaceae, and is considered an important medicinal and edible plant in China with both medicinal and ornamental uses (Zhao et al., 2017). For thousands of years, its stems have been used as ingredients in herbal medicines, teas, and soups, and have proven to be effective in treating diseases, strengthening the body, improving vision, promoting fluid secretion, and improving immunity (Bao et al., 2001; Xing et al., 2013). Nevertheless, because of its unique growing environment, slow growth rate, over-exploitation and environmental degradation, wild *D. huoshanense* is scarce and even endangered. In addition, because of its high medicinal value, *D. huoshanense* has a great demand in the Chinese herbal medicine market.

The chemical constituents of *D. huoshanense* are rich, mainly including polysaccharides, alkaloids, flavonoids, phenols, coumarins, terpenoids, and phenyl compounds (Ng et al., 2012). Polysaccharides, alkaloids, and flavonoids are considered to be the main active components of *D. huoshanense* with important pharmacological effects. This makes it important to understand how they are synthesized. *D. huoshanense* is receiving more and more attention due to its unique medicinal value. Currently, *D. huoshanense* research is mainly focused on the mechanism of synthesis of active ingredients (Luo et al., 2008), identification of the structure (Li et al., 2014), biological activity (Li et al., 2015), and pharmacological effects (Tian et al., 2013). It is noteworthy that the research on active ingredients mainly involves polysaccharides and alkaloids (Yu et al., 2017; Liu et al., 2020), while the research related to flavonoids is relatively rare. In *D. huoshanense*, flavonoids are one of the most important secondary metabolites, and they play a major role in the physiological and developmental processes. Flavonoids have been reported to possess a number of biological properties such as antioxidant activity, antitumor activity, antiviral activity, anti-inflammatory properties, and so on (Anand David et al., 2016).

According to studies, the accumulation of secondary metabolites in plants is influenced by various factors including growth and development, temperature, light and water (Li et al., 2020). As a perennial herb, the stem of *D. huoshanense* is one of the main organs for it to accumulate important medicinal components. The growth year can remarkably affect the accumulation of active ingredients in stem herbs (Yang et al., 2018). For example, the content of most saponins in the roots of 1–3 year-old *Panax notoginseng* increased with age (Jia et al., 2013). The content of ginsenosides in the roots and root-hairs of one to five aged *P. ginseng* was positively correlated with their growth years (Shi et al., 2007). However, not all secondary metabolite levels increase with the growth age of the plant. Bai et al. (2020) studied the content of eight active ingredients in the roots of *Scutellaria baicalensis* from 1 to 15 years and found that the trend of the content of four major medicinal ingredients showed a “V” type. In other words, 2-, 3-, and 15-year old *S. baicalensis* have a higher content of medicinal components than 4- and 5-year old ones. Zhu et al. (2014) found that the amount of triterpenoid in *Codonopsis lanceolata* decreased with age. In conclusion, the effect regarding the growth year on the accumulation of secondary metabolites in plants is complex. Therefore, as a valuable Chinese medicine, it is necessary to study the effect of growth year on the content of its secondary metabolites in *D. huoshanense*.

Currently, transcriptomics, which gives rapid access to genetic information, is widely used to study the molecular regulatory mechanisms of the biosynthetic pathways of active ingredients in different medicinal plants. The application of metabolomics is helping researchers understand the synthesis and accumulation of medicinal components in medicinal plants, and research mainly focused primarily on improved breeding, metabolite identification, disease resistance, and medicinal value (Zhou et al., 2017; Afsheen et al., 2018; Scossa et al., 2018; Hasanpour et al., 2020). Therefore, the combination of transcriptomics and metabolomics has been successfully used to identify key genes and metabolites in a variety of medicinal plants, including *Ginkgo biloba* (Guo et al., 2020), *Coriandrum sativum* (Wu et al., 2021), and *Salvia miltiorrhiza* (Danshen) (Zhan et al., 2019), which has broadened the development of biosynthetic pathways of active ingredients in plants. According to a study on the regulation of color in *D. officinale*, dihydroflavonol was regulated by flavanone 3-hydroxylase (F3H) and leucoanthocyanidin dioxygenase (LDOX), which ultimately resulted in color change (Zhan et al., 2020). Zhao et al. (2015) found that *CYP450* and *MDR* genes are involved in the biosynthesis and transport of coumarin. An analysis of the transcriptome and metabolome of *Dendrobium* used by Liu et al. (2019) led them to hypothesize that 4CL1, *CYP73A*, and *CYP75B1* structural genes are key target genes that play a major role in regulating rutin biosynthesis. Although the biosynthetic pathways of flavonoids have been well studied in plants, such as *Hordeum vulgare* (Shoeva et al., 2016), *Ginkgo biloba* (Meng et al., 2019), and navel orange (Zhang et al., 2021), the pathways of flavonoid biosynthesis in *D. huoshanense* stems are unknown and need further investigation.

Therefore, in this study, we investigated the differential gene expression related to the flavonoid synthesis pathway in the stem tissues of *D. huoshanense* using transcriptomics, compared the differential metabolites in the stems of *D. huoshanense* at different growth years using metabolomics, and screened the key candidate genes and metabolites of flavonoid biosynthesis pathway by combining metabolomics and transcriptomics analysis. The results provide new insights into the development of active medicinal ingredients of *D. huoshanense*.

## Materials and Methods

### Plant Materials

*Dendrobium huoshanense* was artificially cultivated and collected in the greenhouse of Anhui Tongjisheng Biotechnology Co., Ltd. The samples’ cultivation conditions were consistent with our earlier study (Yuan et al., 2018). As the research object, stems of *D. huoshanense* were obtained from 1-year (1Dh_1, 1Dh_2, 1Dh_3), 2-year (2Dh_1, 2Dh_2, 2Dh_3), 3-year (3Dh_1, 3Dh_2, 3Dh_3), and 4-year (4Dh_1, 4Dh_2, 4Dh_3) plants, respectively. A part of materials was frozen in liquid nitrogen at −80°C to extract RNA and metabolites. The other part was washed and dried before being analyzed for total alkaloids and total flavonoids. Furthermore, all experiments in three biological replicates were conducted in the present study.

### Determination of Total Flavonoid and Total Alkaloid Contents

The content of total flavonoids in the stem of *D. huoshanense* was determined using AlCl_3_ colorimetry method (Bouyahya, 2016). Firstly, 0.02 g of dried and crushed powder of the stem of *D. huoshanense* was weighed. The powder was dissolved in 2 mL of 60% (v/v) ethanol and then extracted by ultrasonication for 2 h with the temperature set to 60°C. The filtrate was centrifuged at 10,000 rpm for 10 min, and the supernatant was collected. Next, 30 μl of 5% (w/w) NaNO_2_ was added to 540 μl of the supernatant and mixed well. After 6 min, added 30 μl of 10% (w/w) AlCl_3_ to the mixture and mixed well. 5 min later, 400 μl 1 mol/L NaOH was added to the mixture. Finally, the mixture was left at room temperature for 15 min and the absorbance was measured at 510 nm. The flavonoid content was determined using a standard graph of rutin, and the results were expressed in mg⋅g^–1^ of rutin equivalent. The total alkaloid content was determined by the colorimetric method using acid dyes and a dendrobine standard (Chan et al., 2007).

### Widely Targeted Metabolomics Profiling

We conducted widely targeted metabolomics analyses on samples with three biological duplicates for each growth year to explore metabolite changes among different growth years of *D. huoshanense* stem. Stem samples were sent to Metware Biotechnology Ltd for metabolite analysis (Wuhan, China). For metabolic data analysis, the Analyst 1.6.3 program was used. To identify the differential metabolites, the differences between the metabolites of the two samples were maximized using OPLS-DA (Orthogonal projections to latent structures-Discriminant Analysis). The derived Variable Importance in Projection (VIP) of the OPLS-DA model for multivariate analysis was used to perform a preliminary screening of differential metabolites based on the OPLS-DA results (Worley and Powers, 2016). The differential metabolites for the following step of analysis in our investigation were fold change ≥ 2 and foldchange ≤ 0.5, VIP ≥ 1. We conducted principal component analysis (PCA) to analyze the accumulation of *D. huoshanense* metabolites in different growth years using R package. The data was normalized, and all samples were analyzed using a cluster heatmap that was created.

### RNA Extraction, Illumina Sequencing, and Differentially Expressed Genes

Total RNA was isolated from Omni Plant RNA Kit (CWBIO, China) from *D. huoshanense* stems and tested for quality. The TruSeq™ RNA sample preparation kit (Illumina, United States) was used according to the manufacturer’s instructions, while cDNA libraries were generated using mRNA from each sample. The filtered reads were mapped to the reference genome with the HISAT2 software^1^ (Pertea et al., 2016). Fragments Per Kilobase Million (FPKM) values have been used to evaluate gene expression. The DESeq2 has been utilized to discover genes (DEGs) (Love et al., 2014), and the filter criterion was | log2(fold change)| > 1, with the *p*-value < 0.05. To further understand the function and important pathways of DEGs, TopGO and clusterProfilers were utilized for enriching all DEGs in Gene Ontology (GO) and the Kyoto Encyclopedia of Genes and Genomes (KEGG).

### Gene Co-expression Network Construction

In order to further investigate genes that are significantly linked to the *D. huoshanense* features (years and medicinal components), the R software WGCNA packet has generated a weighted gene co-expression network (Langfelder and Horvath, 2008). To calculate the soft β threshold value, the soft β threshold was chosen and co-expression modules were generated with the Soft Threshold selector function (Xiong et al., 2018). By computing the adjacent matrix on a soft threshold, the topological overlap matrix (TOM), which can reflect the similarity of the co-expression relationship between the two genes was further developed (Yip and Horvath, 2007). Finally, hierarchical clustering approach is employed to produce hierarchical clustering tree of DEGs (Zhan et al., 2015). Hub genes for strongly connected genes that have a high connection level in the co-expression module are widely employed (Zhu et al., 2019; Zhang A. et al., 2020). The top 20 genes with the strongest relationship have been identified as hub genes, according to the size of the module. The genes in these modules were subsequently investigated (Shannon et al., 2003). We have utilized Cytoscape (v.3.6.1) to construct and visualize a gene-gene interaction network.

### Integrated Analysis of Transcriptome and Metabolome

According to the metabolite content and gene expression value in the stem of *D. moniliforme* at different growth years, the DEGs and the DAMs of flavonoid biosynthesis pathway in each comparison group were analyzed. First, pathway analysis was used to analyze the DEGs and DAMs related to flavonoid biosynthesis. Moreover, in order to better understand the relationship between transcriptome and metabolome, DEGs, and DAMs were mapped to the KEGG pathway database to obtain their common pathway information.

### Real-Time Quantitative PCR Validation

The following 11 DEGs were chosen for qRT-PCR analysis: *LOC110101536, LOC110107833, LOC110095936, LOC110113 268, LOC110115941, LOC110103762, LOC110106061, LOC110 097028, LOC110097219, LOC110107557*, and *LOC110114894*. The approach used 10 ng of cDNA extracted from the stems of each growth year. The experiment was performed on an ABI 7500 Real-time PCR system (Applied BioSystems, United States) using SYBR Premix Ex Taq (Takara, Japan) according to the manufacturer’s protocol. The *Actin* was utilized as reference genes. The 2^–ΔΔCT^ technique was used to calculate relative expression levels (Livak and Schmittgen, 2001). Supplementary Table 1 listed the primers that were used in this study.

## Results

### Determination of Flavonoids and Alkaloids Contents and Identification of Differentially Expressed Genes

**FIGURE 1:**
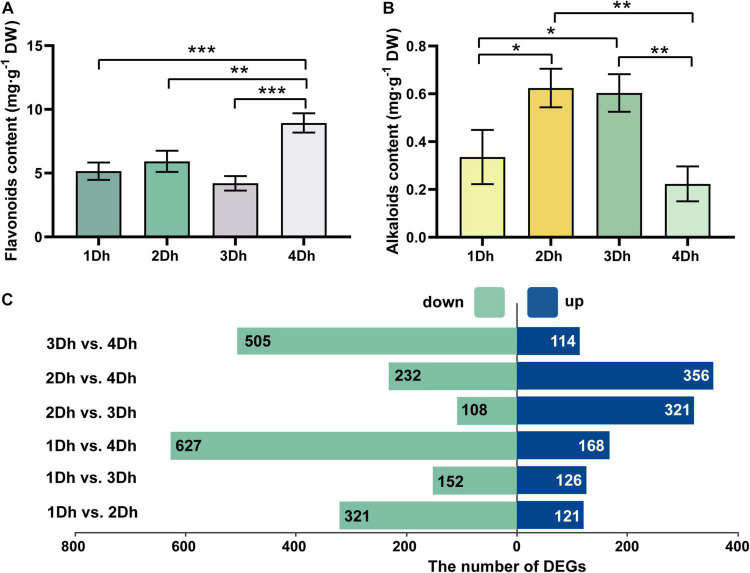
Determination of total flavonoids and alkaloids in the stem of *D. huoshanense* and DEGs in different comparison groups. Error bars represent standard deviations. Asterisks “*” mean statistical differences of the same index between different growth years, and *p* < 0.05 is a significant difference (*p < 0.05, **p < 0.01, and ***p < 0.001). **(A)** Content of total flavonoids in stems of *D. huoshanense* with different growth years. **(B)** Contents of total alkaloids in stems of *D. huoshanense* with different growth years. **(C)** The number of DEGs in different comparison groups.

The variations of flavonoid and alkaloid contents at different growth years (1, 2, 3, and 4Dh) are shown in Figures 1A,B. The results showed that there were some differences in flavonoid and alkaloid contents in the stems of *D. huoshanense*. There was an increase in flavonoid content from 1 to 2Dh, a gradual decline from 2 to 3Dh, but then a significant increase from 3 to 4Dh and a peak at 4Dh, with flavonoid content was 8.94 mg⋅g^–1^ at 4Dh. Based on statistical analysis, the flavonoid content of *D. huoshanense* of 4-year-old plants was significantly different from that of *D. huoshanense* of other years. The results indicate that the fourth year is the optimal period for flavonoid accumulation. Also, we measured the alkaloid content of stems of *D. huoshanense* from different growth years. There was a significant increase in alkaloid content from the first year to the second, a slight decrease from the second to the third year, and a very significant decrease from the third to the fourth year. The results indicate significant differences between the alkaloid contents of 4-year-old *D. huoshanense* and those of 2-year-old and 3-year-old *D. huoshanense*. Generally, the highest alkaloid content of 0.62 mg⋅g^–1^ DW was found in 2-year-old *D. huoshanense* while the lowest alkaloid content of 0.22 mg⋅g^–1^ DW was found in 4-year-old *D. huoshanense*. On average, the second and third years are the best times for alkaloids to accumulate.

In this study, 12 cDNA libraries (1Dh_1, 1Dh_2, 1Dh_3 for 1-year-old *D. huoshanense* stems, 2Dh_1, 2Dh_2, 2Dh_3 for 2-year-old *D. huoshanense* stems, 3Dh_1, 3Dh_2, 3Dh_3 for 3-year-old *D. huoshanense* stems and 4Dh_1, 4Dh_2, 4Dh_3 for 4-year-old *D. huoshanense* stems) were prepared and analyzed. We obtained 694,154,682 raw reads from the 12 libraries in total. We filtered out the low-quality sequences and obtained 627,197,798 clean reads, with average Q20 and Q30 values of 97.77 and 94.25%, respectively. The clean reads were assembled into contigs using Trinity, the contigs were clustered and partially assembled to obtain transcripts, and finally the dominant transcripts were selected as genes. We obtained 20, 364 genes ranging in length from 100 to 16,928 base pairs (bp). Sequence data were submitted to the Genome Sequence Archive (Genomics, Proteomics, and Bioinformatics 2021) (accession number: CRA005817).

We constructed six comparison groups to demonstrate the differences in transcriptome of *D. huoshanense* under different growth years: 1Dh vs. 2Dh, 1Dh vs. 3Dh, 1Dh vs. 4Dh, 2Dh vs. 3Dh, 2Dh vs. 4Dh, 3Dh vs. 4Dh, and screened with *p* value < 0.05 and | log2(FC)| > 1 for the total number of differentially expressed genes (DEGs) was 1802. Among them, the number of up-regulated and down-regulated genes for each comparison group is represented in Figure 1C. There were a total of 1802 DEGs identified in the six comparison groups of 1Dh vs. 2Dh, 1Dh vs. 3Dh, 1Dh vs. 4Dh, 2Dh vs. 3Dh, 2Dh vs. 4Dh, and 3Dh vs. 4Dh. The greatest number of DEGs were identified between 1 and 4Dh, among which 168 up-regulated DEGs and 627 down-regulated DEGs. Compared with 2Dh, 1Dh contained the least number of DEGs, 126 being up-regulated and 152 being down-regulated. According to the above results, there were significant differences in DEGs between the two groups.

### Gene Ontology Enrichment Analysis of the Differentially Expressed Genes

**FIGURE 2:**
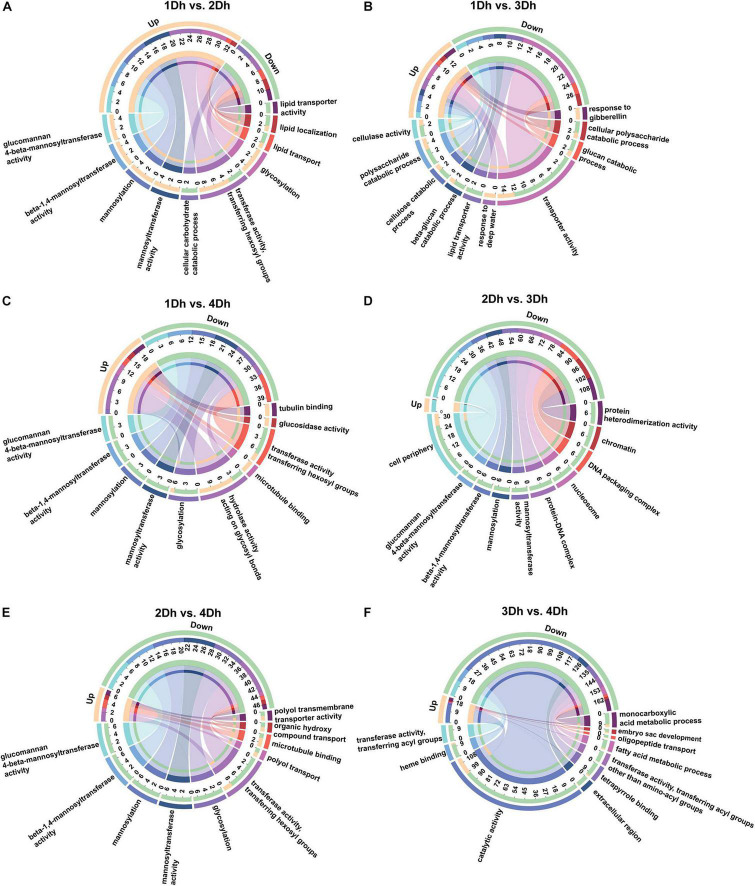
GO enrichment circos plot of DEGs. The upper half-circle indicates the number of up and down regulated DEGs in each comparison group, orange indicates up-regulation and green indicates down-regulation. The outermost circle of the lower half-circle indicates the GO terms, and different colors represent different GO terms; the inner circle indicates the number of up- and down-regulated DEGs in each GO term. The top 10 GO terms that are significantly enriched are shown in the figure. The chords in the figure point from up- and down-regulated genes to GO terms, and the thickness of the chords indicates the abundance of up- and down-regulated genes in each GO term. **(A)** 1Dh vs. 2Dh. **(B)** 1Dh vs. 3Dh. **(C)** 1Dh vs. 4Dh. **(D)** 2Dh vs. 3Dh. **(E)** 2Dh vs. 4Dh. **(F)** 3Dh vs. 4Dh.

Gene ontology enrichment analysis classified all DEGs into Biological Process (BP), Cellular Component (CC) and Molecular Function (MF) categories. The 1802 DEGs of all samples were annotated to 1468 GO terms; among them, the highest percentage of BP (59.81%), followed by MF (29.02%) and CC (11.17%). As shown in Figure 2, among the top 10 significantly enriched terms, the categories with higher enrichment in each comparison group were different, and the number of down-regulated DEGs was higher than the number of up-regulated DEGs in all comparison groups except 1Dh vs. 2Dh. A total of 6 terms were highly enriched in the top 10 GO terms enriched in the 1Dh vs. 2Dh, 1Dh vs. 4Dh, 2Dh vs. 3Dh, and 2Dh vs. 4Dh groups, and most of them were involved in these 4 groups, including “Glucomannan 4-beta-mannosyltransferase activity” (GO:0047259), “Beta-1,4-mannosyltransferase activity” (GO:0019187), “Mannosyltransferase activity” (GO:0000030), “Transferase activity, transferring hexosyl groups” (GO:0016758), “Mannosylation” (GO:0097502), and “Glycosylation” (GO:0070085), but the number of enriched DEGs differed among the comparison groups, indicating that transferase activity has an important position in the molecular function. Notably, the cellular component (CC) category was significantly enriched in 1Dh vs. 4Dh, “Cell periphery” (GO:0071944, 32 DEGs) was the largest of the cellular classes, while the CC class was less enriched in the rest of the comparison groups.

Among the top 10 GO terms, the comparison groups 1Dh vs. 3Dh and 3Dh vs. 4Dh differed greatly from the other comparison groups. In 1Dh vs. 3Dh, a total of 21 DEGs were enriched in biological processes, which were mainly concentrated in catabolic processes, respectively, including “Polysaccharide catabolic process” (GO:0000272, 5 DEGs), “Cellulose catabolic process” (GO:0030245, 3 DEGs), “Beta-glucan catabolic process” (GO:0051275, 3 DEGs), “Glucan catabolic process” (GO:0009251, 3 DEGs), “Cellular polysaccharide catabolic process” (GO:0044247, 3 DEGs); additionally, 21 DEGs were also enriched for molecular functional types, 71.42% of DEGs were mapped to transporter activity and the remainder was mapped to “Cellulase activity” (GO: 0008810, 3 DEGs), “Lipid transporter activity” (GO:0005319, 3 DEGs). Comparing 3Dh and 4Dh, there were four terms enriched by BP and more DEGs enriched by metabolic process. In summary, the GO terms significantly enriched in the six comparison groups in the biological process category were “catabolic process” and “metabolic process.” “Binding” and “Transferase activity” were significantly enriched in the molecular function category. For cell formation, the terms enriched by different comparison groups were significantly different, mainly including “Cell periphery,” “Protein-DNA complex,” “Protein-DNA complex,” “Nucleosome,” and “Chromatin,” *etc*.

### Kyoto Encyclopedia of Genes and Genomes Enrichment Analysis of the Differentially Expressed Genes

**FIGURE 3:**
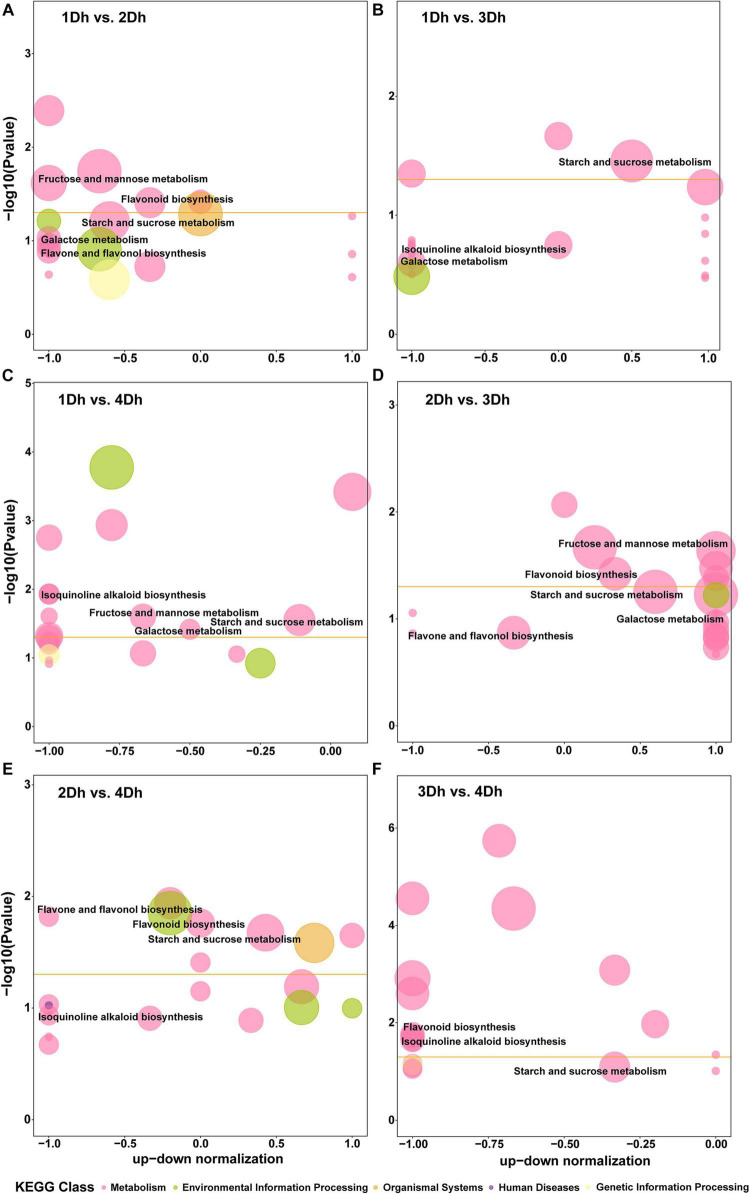
KEGG pathway enrichment bubble diagram. The horizontal axis is the up-down normalization and the vertical axis is the –log_10_ (*P*-value). The size of the bubble represents the number of the target DEGs and the color of the bubble represents different KEGG classes. The threshold line in orange is *P-*value = 0.05. **(A)** 1Dh vs. 2Dh. **(B)** 1Dh vs. 3Dh. **(C)** 1Dh vs. 4Dh. **(D)** 2Dh vs. 3Dh. **(E)** 2Dh vs. 4Dh. **(F)** 3Dh vs. 4Dh.

Based on the KEGG annotation results, 1802 DEGs in all samples were mapped to 99 KEGG pathways in 6 categories (level 1), including Cellular Processes (3 pathways), Environmental Information Processing (4 pathways), Genetic Information Processing (15 pathways), Genetic Information Processing (1 pathway), and Metabolism (74 pathways), and Organismal Systems (2 pathways). The Metabolism category had the most pathways and genes, whereas the Genetic Information Processing category had the least. In Figure 3, the top 20 enriched pathways in each comparison group are displayed. A total of 96 DEGs were identified in the 1Dh vs. 2Dh comparison, with the enriched pathways being “Sesquiterpenoid and triterpenoid biosynthesis” (dct00909), “Starch and sucrose metabolism” (dct00500), “Fructose and mannose metabolism” (dct00051), “Glutathione metabolism” (dct00480), “Flavonoid biosynthesis” (dct00941), “Phenylpropanoid biosynthesis” (dct00940), “Plant hormone signal transduction” (dct04075), and “Plant-pathogen interaction” (dct04626). The main enrichment pathways in the other comparison groups were roughly the same as 1Dh vs. 2Dh.

In general, the secondary metabolic pathways were significantly enriched. Secondary metabolism is of great importance to medicinal plants (Fan et al., 2021). The KEGG pathways related to secondary metabolism in each comparison group were mainly focused on flavonoids, alkaloids and polysaccharides, these include “Flavonoid synthesis” (dct00941), “Phenylpropanoid biosynthesis” (dct00940), “Flavonoid and flavonol biosynthesis” (dct00944) pathways involved in flavonoid biosynthesis; “Isoquinoline alkaloid biosynthesis” (dct00950), “Tropane, piperidine and pyridine alkaloid biosynthesis” (dct00960) pathways involved in alkaloid biosynthesis; “Starch and sucrose metabolism” (dct00500), “Fructose and mannose metabolism” (dct00051), “Galactose metabolism” (dct00052) pathways involved in polysaccharide biosynthesis. And these three medicinal components are the main medicinal components of *Dendrobium* species.

### Widely Targeted Metabolomics Analysis and Overall Metabolite Identification

**FIGURE 4:**
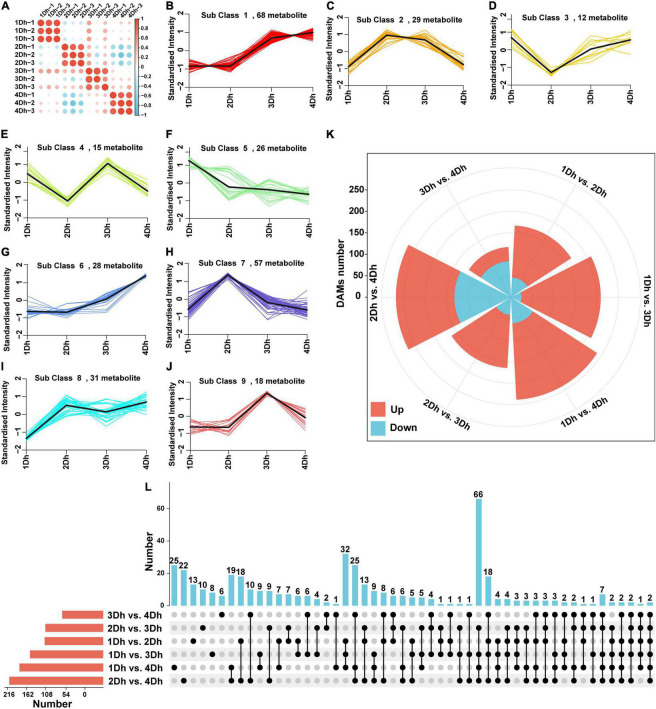
Metabolomic analyses of *D.huoshanense* of different growth years. **(A)** Correlation heatmap of all the samples; **(B–J)** The K means analysis of DAMs. **(K)** Rose diagram of the up- and down-regulated distribution of DAMs. **(L)** UpSet diagram of DAMs in different comparison groups.

In order to evaluate the variation of metabolites in stems of different growth years, a broadly targeted metabolomic analysis was conducted. We obtained a total of 767 metabolites, consisting mainly of amino acids and their derivative (82), phenolic acids (107), nucleotides and their derivatives (51), flavonoids (173), quinones (21), lignans (12), coumarins (11), tannins (4), alkaloids (54), terpenoids (7), organic acids (49), and lipids (101). Clustering heatmap (Figure 4A) analysis between the comparison groups revealed a high degree of similarity between the three biological replicates of each treatment, indicating the high reliability of the metabolomics data. To understand the trends of metabolites at different growth years, 284 metabolites were clustered using a K-means clustering method. Based on the expression patterns of different metabolites, nine clusters were obtained (Figures 4B–J). Noteworthy, alkaloids were mainly found in cluster 2, and the trend was that the expression of metabolites was higher in 2Dh and 3Dh than in 1Dh and 4Dh, which was consistent with the change in alkaloid content.

Additionally, we examined the variation of DAM in *D. huoshanense* in different growth years (Figure 4K), and 428 DAMs were identified. Among all six comparison groups, the greatest number of DAMs (268) was detected in 2Dh vs. 4Dh, and the smallest number of DAMs (117) was detected in 3Dh vs. 4Dh; where the number of up- and down-regulated DAMs in each group is presented in Figure 4K. In all groups except 3Dh vs. 4Dh, the number of up-regulated DAMs was greater than the number of down-regulated DAMs. In addition, unique and shared metabolites were identified (Figure 4L). Interestingly, the number of shared DAMs was highest between 1Dh vs. 3Dh, 1Dh vs. 4Dh, 2Dh vs. 3Dh, and 2Dh vs. 4Dh, and two metabolites were commonly detected in all six groups.

**FIGURE 5:**
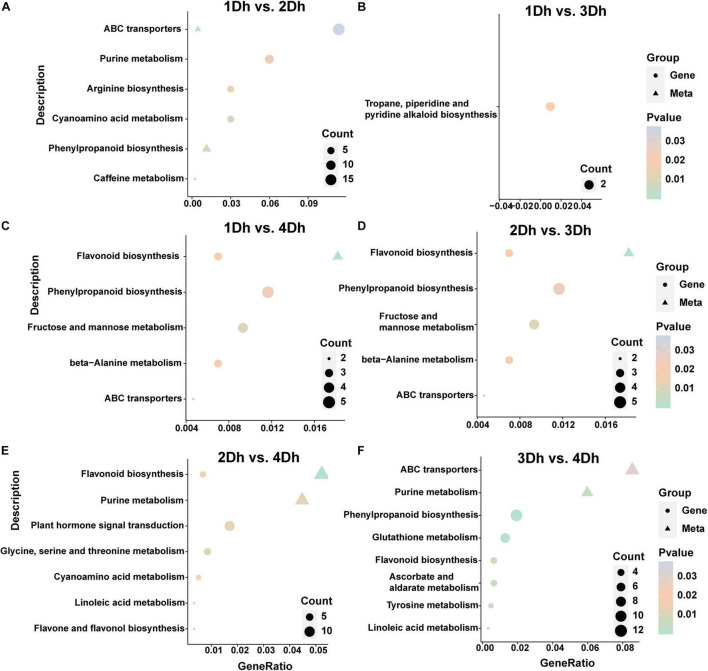
Integrated with DEGs and DAMs of KEGG pathway. **(A)** 1Dh vs. 2Dh. **(B)** 1Dh vs. 3Dh. **(C)** 1Dh vs. 4Dh. **(D)** 2Dh vs. 3Dh. **(E)** 2Dh vs. 4Dh. **(F)** 3Dh vs. 4Dh.

A KEGG enrichment analysis was performed to further reveal the potential role of DAMs. The top 20 enriched pathways in each of the six comparison groups were shown in Figure 5. The highly enriched pathways may be the key to the variation of metabolites in different growth years of *D. huoshanense*. The results showed that the enriched pathways were similar in 1Dh vs. 2Dh and 3Dh vs. 4Dh, with the main enriched pathways of DAMs being “Metabolic pathways” (dct01100), “Biosynthesis of secondary metabolites” (dct01110), and “ABC transporters” (dct02010). Also, DAMs involved in “Purine metabolism” (dct00230), “Biosynthesis of amino acids” (dct01230) was noted in the comparison of 1Dh vs. 3Dh, 2Dh vs. 3Dh and 2Dh vs. 4Dh. In particular, “Flavonoid biosynthesis” (dct00941) and “Flavone and flavonol biosynthesis” (dct00944) were significantly enriched in 1Dh vs. 3Dh, 1Dh vs. 4Dh, 2Dh vs. 3Dh, and 2Dh vs. 4Dh, suggesting that flavonoid biosynthesis is a key enrichment pathway.

### An Integration Transcriptomic and Metabolic Analysis

A better understanding of gene regulation in metabolic processes can be gained by combining transcriptomics and metabolomics analysis. To examine the relationship between genes and metabolites, we mapped DEGs and DAMs in each comparison group to the KEGG pathway (Supplementary Figure 1). In 1Dh vs. 2Dh, 30 DEGs and 78 DAMs were mapped to 22 KEGG pathways, with “Phenylpropanoid biosynthesis” (dct00940) and “ABC transporters” (dct02010) being the major enriched pathways. In 2Dh vs. 4Dh, 69 DEGs and 139 DAMs were compared to 32 KEGG pathways, of which 7 were significantly enriched including “Flavonoid biosynthesis” (dct00941), “Flavone and flavonol biosynthesis” (dct00944). In 3Dh vs. 4Dh, 46 DEGs and 36 DAMs were mapped to 13 KEGG pathways, “ABC transporters” (dct02010), “Purine metabolism” (dct00230), “Phenylpropanoid biosynthesis” (dct00940), “Flavonoid biosynthesis” (dct00941) were these DEGs and DAMs in the most representative pathways. Notably, in 1Dh vs. 3Dh, 27 DEGs and 34 DAMs were distributed to 20 KEGG pathways, but only “Tropane, piperidine and pyridine alkaloid biosynthesis” (dct00960) was significantly enriched, including 2 DEGs (*LOC110101810; LOC110097083*) and two DAMs (pme0026, mws0258), suggesting that these two genes and these two metabolites may have an important impact in the alkaloid synthesis pathway.

Furthermore, it is interesting to note that 1Dh vs. 4Dh (113 DEG and 91 DAM) and 2Dh vs. 3Dh (52 DEG and 86 DAM) were mapped to 35 and 22 KEGG pathways, respectively, and the pathways significantly enriched in both groups were identified, mainly including “Fructose and mannose metabolism” (dct00051), “Phenylpropanoid biosynthesis” (dct00940), and “Flavonoid biosynthesis” (dct00941), indicating that the biosynthesis of flavonoids and polysaccharides plays a significant role in both group. And, among the 3 DEGs and 13 DAMs that were simultaneously enriched in the “Flavonoid biosynthesis” pathway in 1Dh vs. 4Dh, 1 DEG (*LOC110113268*) and 9 DAMs (mws0064, mws0914, mws1094, mws1033, pmp000571, pme0088, mws0463, pme0376, mws0044, pme3475, mws0920, pme1201, and pme2960) were located in the flavonoid synthesis pathway; in 2Dh vs. 3Dh, among the 3 DEGs and 14 DAMs both enriched in the “Flavonoid biosynthesis” pathway, 2 DEGs (*LOC110097028, LOC110095936*) and 10 DAMs (pme0376, mws1033, mws1094, mws1179, mws0064, pme2960, pmp000571, pme1201, pme0088, and mws0044) were located in the flavonoid synthesis pathway (Figure 5). Overall, “Phenylpropanoid biosynthesis” (dct00940), “Flavonoid biosynthesis” (dct00941), “Flavone and flavonol biosynthesis” (dct00944) were more abundant in these six comparative groups, indicating that flavonoid biosynthesis is crucial in *D. huoshanense*.

### Metabolic Changes and Transcriptomic Regulation Associated With Flavonoid Biosynthesis

**FIGURE 6:**
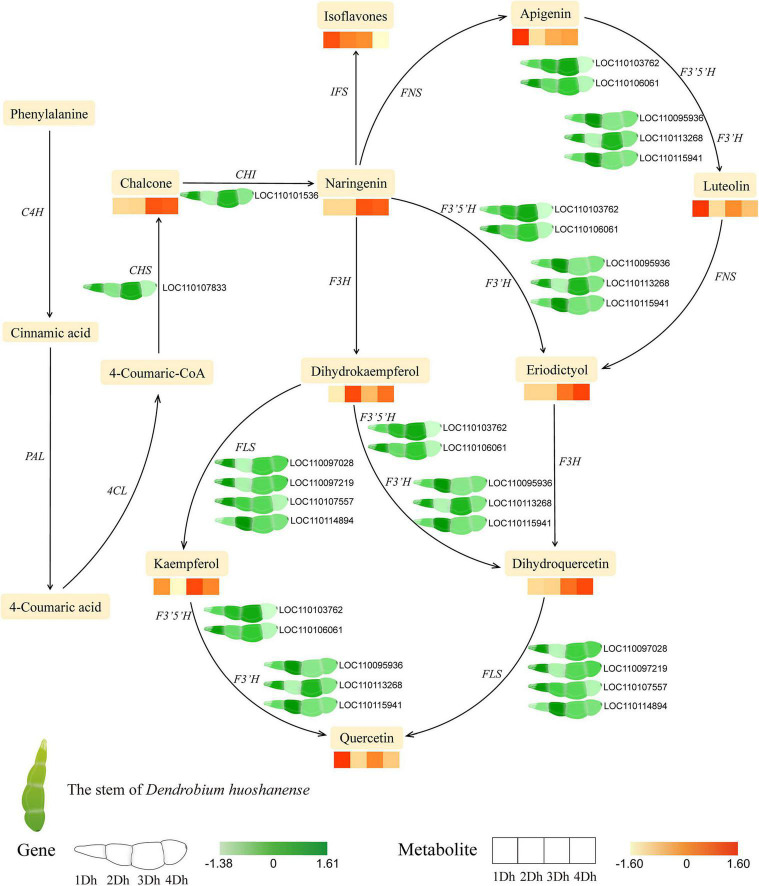
The pathway associated with flavonoid biosynthesis in *D. huoshanense*. The expression heatmap of the key metabolites and transcripts for flavonoid synthesis between four groups was shown by the colored cell on the bottom. Key enzyme gene abbreviation: *PAL*, phenylalanine ammonia lyase; *4CL*, 4-coumarate: CoA ligase; *C4H*, cinnamate 4-hydroxylase; *CHS*, chalcone synthase; *CHI*, chalcone isomerase; *F3H*, flavonoid 3-hydroxylase; *F3’H*, flavonoid 3’-hydroxylase; *F3’5’H*, flavonoid 3’,5’-hydroxylase; *FNS*, flavone synthase; *FLS*, flavonol synthase; *AOMT*, flavonoid O-methyltransferase.

According to the results of KEGG enrichment analysis of DEGs and DAMs, “Phenylpropanoid biosynthesis,” “Flavonoid biosynthesis,” “Flavone and flavonol biosynthesis” were significantly enriched in each comparison group. Therefore, we explored the changes of genes and metabolites involved in flavonoid biosynthesis in different growth years (Figure 6). In this study, we comprehensively analyzed the expression patterns of key enzyme genes (*PAL*, *4CL*, *ANS*, *C4H*, *CHI*, *CHS*, *DFR*, *F3’5’H*, *F3H*, *F3’H* and *FLS*, *etc*.) in flavonoid biosynthesis, in which a total of 11 DEGs were identified based on transcriptome data DEGs, including one *CHS* (*LOC110107833*), one *CHI* (*LOC110101536*), two *F3’5’H* (*LOC110103762, LOC110106061*), three *F3’H* (*LOC110095936, LOC110113268, LOC110115941*), and 4 *FLS* (*LOC110097028, LOC110097219, LOC110107557, LOC110114894*). To verify the reliability of transcriptome data, we evaluated the expression of these genes using qRT-PCR. The results showed that although there were some differences, the trend of qRT-PCR results was basically consistent with RNA-Seq data, which further proved the reliability of sequencing results (Supplementary Figure 2A–D). In Supplementary Figure 2E, we analyzed the correlation between the qRT-PCR data and transcriptome data. As shown in the figure, *r* = 0.804, indicating that there was a strong correlation between the qRT-PCR and transcriptome data. This again confirmed that our transcriptome data were valid.

In addition, we successively analyzed the expression of key metabolites. In total, 46 DAMs were identified in the flavonoid biosynthetic pathway, mainly including 2 Chalcones, 3 Naringenin, 23 Flavonoid (4 Eriodictyol, 11 Apigenin, and 8 Luteolin), 16 Flavonols (2 Dihydrokaempferol, 4 Kaempferol, 1 Dihydroquercetin, 9 Quercetin) and 2 isoflavones. Similarly, there was some variation between metabolite expression levels, with Chalcone, Naringenin Dihydroquercetin, and Eriodictyol were more expressed in 3Dh and 4Dh than in 1Dh and 2Dh, Kaempferol, Quercetin, Apigenin, and Luteolin were more expressed in 1Dh and 3Dh, and the trend of Dihydrokaempferol expression showed higher in 2Dh and 4Dh than in 1Dh and 3Dh, while Isoflavones showed a gradually decreasing trend. Overall, the expression trends of most genes were basically consistent with the trends of their downstream metabolites.

### Weighted Gene Co-expression Network Analysis

**FIGURE 7:**
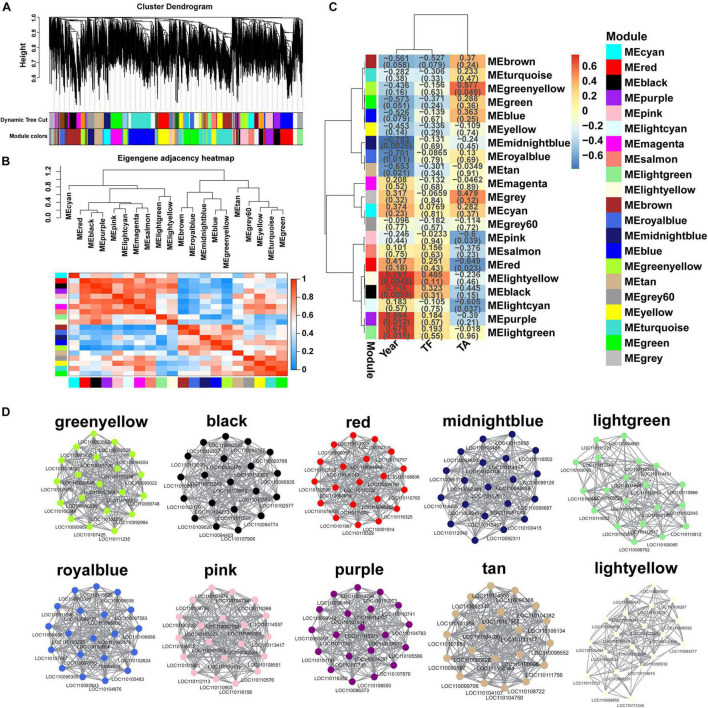
Weighted gene co-expression network of *D. huoshanense*. **(A)** Clustering dendrogram of DEGs, with dissimilarity based on the topological overlap, together with assigned module colors. The clustered branches represent different modules, and each line represents one DEG. **(B)** The heatmap of connectivity of eigengenes. **(C)** Module-trait associations. Each row corresponds to a module characteristic gene (eigengene), and each column corresponds to a trait. Each cell contains a corresponding correlation coefficient and *p*-value. **(D)** Visualization of network relationships of hub genes in 10 modules.

In order to get a comprehensive understanding of the relationship between genes in each growth year of *D. huoshanense*, WGCNA analysis was performed with 1802 DEGs as the source data, and 21 modules were identified (Figure 7A), namely black, blue, brown, cyan, green, greenyellow, gray, gray, lightcyan, lightgreen, lightyellow, magenta, midnightblue, pink, purple, red, royalblue, salmon, tan, turquoise, and yellow. The total number of genes in each of these modules varied widely, ranging from 33 DEGs in the royalblue module to 244 DEGs in the turquoise module. To detect the interaction relationship between these gene modules, we performed neighbor-joining heatmap for these modules (Figure 7B), and the results showed that the correlation and neighbor-joining between the modules were good. Next, we analyzed the correlation between the traits (Year, total flavonoids = TF), total alkaloids = TA) and the expression pattern of each module (Figure 7C). The results showed that 10 modules were highly correlated with Year, TF, and TA, namely greenyellow, black, red, midnightblue, lightgreen, royalblue, pink, purple, tan, and lightyellow, and the other modules were less correlated with the traits. We analyzed in depth the changes in gene expression in these highly correlated modules and the relationship between modules and traits.

In this study, we analyzed key gene modules and hub genes related to flavonoid synthesis. For finding the key genes, we identified 20 hub genes in each of greenyellow, black, red, midnightblue, lightgreen, royalblue, pink, purple, tan, and lightyellow modules, and constructed 10 gene regulatory networks (Figure 7D) to visualize the association between genes. Our results demonstrated that the hub genes were closely connected between each other and the connectivity was strong. It is noteworthy that we identified two genes involved in the flavonoid synthesis pathway among these 200 hub genes, specifically one *F3’H* gene (*LOC110095936*) in the royalblue module and one *FLS* gene (*LOC110107557*) in the tan module, suggesting that the royalblue and tan modules play an important role in flavonoid accumulation and regulate the biosynthesis of flavonoids, further suggesting that *F3’H* and *FLS* genes are the key to flavonoid accumulation in *D. huoshanense*.

## Discussion

As an important medicinal plant in China, the study of the medicinal value of *D. huoshanense* has gained increasingly importance. At present, more and more studies have identified the active components of *D. huoshanense*, however, most of these studies have focused on polysaccharides and mainly investigated their structure and biological activity of polysaccharides (Li et al., 2014; Liu et al., 2020). It remains to be determined how flavonoid accumulation takes place in *D. huoshanense*, as well as the mechanisms by which it occurs. We identified the genes and metabolites of *D. huoshanense* based on transcriptomic and metabolomic data in this study, and thereby gained insights into the accumulation of flavonoids in *D. huoshanense* stems. As a result of analyzing the expression of flavonoid metabolites in different growth years, we noticed different accumulation patterns of metabolites involved in flavonoid biosynthesis. In contrast, most metabolites were higher expressed in 3Dh and 4Dh, suggesting that appropriate regulation of *D. huoshanense* in different growing years could improve the medicinal efficacy of flavonoids. We identified a total of 11 DEGs and 46 DAMs in the four growth years using metabolomics and transcriptomics data. In the metabolomics analysis, Chalcones, Naringenin, Eriodictyol, Dihydrokaempferol, Kaempferol, Dihydroquercetin, and other important secondary metabolites accumulated significantly and showed high expression at 3Dh and 4Dh. On the transcriptomic level, most *CHS*, *CHI*, *F3’H*, *F3’5’H*, and *FLS* genes were expressed at higher levels in 3Dh. Co-expression of these genes may contribute to flavonoid synthesis and may be responsible for the increased accumulation of flavonoids in the stems of *D. huoshanense*.

Under different growth years, we examined the changes in transcriptome and metabolome of *D. huoshanense* stems. As expected, the genes and metabolites relating to flavonoids changed significantly in *D. huoshanense* at different growth years. By examining the flavonoid synthesis pathway, it is evident that the biosynthesis of phenylpropanoids is a complex and important process. *PAL*, *C4H*, and *4CL* are catalyzed in the initial steps of flavonoid synthesis and provide the basis for subsequent steps (Falcone Ferreyra et al., 2012). Phenylalanine, the upstream of flavonoids, is first converted to 4-Coumaric-CoA catalyzed by PAL, C4H and 4CL. Subsequently, 4-Coumaric-CoA is converted to naringenin catalyzed by CHS and CHI. While naringenin is the key metabolite in flavonoid synthesis (Figure 6), catalyzed by different enzymes, the naringenin produces three branches, one branch produces flavonoid (eriodictyol, apigenin, and luteolin), the second branch produces flavonols (dihydrokaempferol, kaempferol, dihydroquercetin, and quercetin), and the third branch produces isoflavones, which is in agreement with the previous findings (Gao et al., 2020; Jiang et al., 2020). here have been reports that naringenin possesses a broad range of biological activities, such as anti-oxidant, hepatoprotective, anti-inflammatory, and anticarcinogenic effects (Raso et al., 2001), and the pharmacological potential of flavonoids in *D. huoshanense* needs to be further explored.

In addition, transcriptomic and metabolomic analysis revealed that the genes encoding *CHS* and *CHI* were significantly associated with downstream flavonoid compounds. *CHS* and *CHI* showed higher expression at 3Dh, while their catalytic products chalcone and naringenin also showed higher expression at 3Dh and 4Dh, consistent with the pattern of changes in their downstream metabolites, which provides a basis for flavonoid accumulation and a preliminary product. Indeed, *CHS* and *CHI* have important roles in flavonoid synthesis and are key enzymes for the production of important flavonoids. It has been shown that the expression of *CHI* gene was significantly and positively correlated with flavonoid accumulation in *Lycium chinense* (Guan et al., 2014), *Citrus unshiu* (Wang et al., 2010) and *Ipomoea batatas* (Guo et al., 2015). Similarly, CHS has an enzymatic role in naringenin biosynthesis (Sun et al., 2015) and its expression was significantly correlated with anthocyanin accumulation (Wang et al., 2017), which regulates the petal coloration process (Tai et al., 2014). Thus, based on metabolomic and transcriptomic data, we hypothesized that high expression of upstream genes and metabolites promoted the accumulation of downstream flavonoids. Notably, the analysis of transcriptomic data in this study revealed that three genes, *F3’H*, *F3’5’H*, and *FLS*, underwent significant changes across growth years and catalyzed multiple processes, respectively. For example, FLS catalyzed the conversion of dihydrokaempferol to kaempferol and naringenin to kaempferol. It has been shown that F3’5’H and F3’H catalyze the conversion of apigenin to luteolin, naringenin to eriodictyol, dihydrokaempferol to dihydroquercetin, and kaempferol to quercetin, and most of the *F3’H*, *F3’5’H*, and *FLS* genes show relatively high expression at 3Dh. Therefore, we speculate that the high expression of *F3’H*, *F3’5’H*, and *FLS* may play an important role in the biosynthesis of flavonoids in the stems of *D. huoshanense* and is one of the important reasons for the accumulation of flavonoids. It was reported that Yukiko et al. found by Northern blot analysis that the accumulation of anthocyanins and flavonoids was transcriptionally regulated by genes such as *F3’H*, *F3’5’H* (Yip and Horvath, 2007). Whang et al. (2011) found that *F3’5’H* can regulate the color in perianths of *D. moniliforme*. Wei et al. (2015) similarly found that in *Camellia sinensis*, *F3’H* and *F3’5’H* are key enzymes involved in flavonoid synthesis, affecting the formation of dihydroxylated and trihydroxylated catechins. Similarly, Fang et al. (2013) investigated the changes in FLS and flavonol content during grape fruit development and showed that FLS is the key enzyme for flavonol synthesis and determines the final content of flavonols, and its activity was highly positively correlated with the total flavonol content. These results further confirmed the reliability of our speculation.

We found that flavonoids were significantly accumulated in *D. huoshanense* stems at the third and fourth growth years, and the expression of genes related to flavonoid biosynthesis like *CHS*, *CHI*, *F3’5’H*, and *FLS* showed similar changes. Transcriptomic and metabolomic analyses have also been used to examine the accumulation patterns of major components during growth in diverse species. A study found that flavonoid content decreases significantly with apple fruit development (Xu et al., 2020), whereas it increases significantly during the growth of *Eucommia ulmoides* leaves (Cheng et al., 2019). Anthocyanin content in pink tea increased continuously during the development and little in white teas, primarily as a result of low expression of *FLS* and high expression of *DFR* in pink teas (Zhou et al., 2020). In *Citrus sinensis* flavonoids accumulate at an early stage and decrease at a later stage, probably as a result of the decreased expression of *PAL*, *4CL* and *CHI* genes during fruit development (Zhang et al., 2021). In *Anoectochilus roxburghii* grown in symbiosis with mycorrhizal fungi, up-regulation of *CHS* gene expression at 2, 3, and 4 months of growth resulted in different levels of corresponding flavonoid accumulation (Zhang Y. et al., 2020). The gradual increase in flavonol compounds during flowering in tea tree was associated with a significant up-regulation of *FLS* genes (Shi et al., 2021). This indicates that compounds of different species exhibit different accumulation patterns during growth.

Numerous studies have been reported on the genes involved in the flavonoid synthesis pathway in Dendrobium species, focusing primarily on *D. officinale*. The genes encoding flavonoid biosynthesis are tissue-specific in Dendrobium. Yuan et al. (2020) found that genes controlling flavonoid biosynthesis, such as *LAR*, *DFR*, and *F3H*, were significantly upregulated in *D. officinale* leaves as compared to roots and stems. In *D. officinale*, Wang Z. et al. (2021) found that the expression of genes encoding enzymes involved in flavonoid biosynthesis was different between protocorm-like bodies and leaves. The expression of genes involved in flavonoid biosynthesis in *D. catenatum* from different geographical locations differed, with most of them being highly expressed in the Zhejiang samples (Lei et al., 2018). Meanwhile, the expression of genes involved in flavonoid biosynthesis was differed markedly in different species of *Dendrobium*. The expression of structural genes such as *CHS*, *C4H*, and *F3’H* was higher in *D. denneanum*, which may explain the higher content of flavonoids in *D. denneanum* than in *D. chrysotoxum*, *D. nobile*, and *D. fimbriatum* (Wang Y. et al., 2021).

It is noteworthy that genes are inextricably linked to metabolites; genes generally exhibit consistent changes with downstream metabolites, while high or low expression of key genes, to some extent, results in the accumulation or decrease of metabolites during growth. Consequently, the joint analysis of metabolomics and transcriptomics data is an effective analytical method for elucidating key genes and metabolites in biosynthetic pathways. In this study, we investigated the changes in genes and metabolites involved in the biosynthesis of flavonoids in *D. huoshanense* at different growth years. The results confirmed the increased expression of several genes and the accumulation of metabolites at various stage of growth. Overall, this study provides new insights into the molecular basis for metabolic differences and extends the understanding of transcription and metabolism in *D. huoshanense* stems, thereby providing favorable evidence and directions for further research.

## Conclusion

Despite being an important class of secondary metabolites in *D. huoshanense*, little is known about the biosynthesis and regulation of flavonoids. Our study compared the transcriptomes of stems from four different years of *D. huoshanense* to identify key genes involved in flavonoid biosynthesis, which play a key role in the accumulation of flavonoids. In the combined transcriptome and metabolome analysis, we found that the third year of cultivation may indicate that *D. huoshanense* is accumulating flavonoids, thus requiring special cultivation regulation by growers during this period. In this study, we have provided a new perspective for understanding the regulatory mechanisms that govern the flavonoid biosynthesis in *D. huoshanense*.

## Data Availability Statement

The datasets presented in this study can be found in online repositories. The names of the repository/repositories and accession number(s) can be found below: Genome Sequence Archive with the accession number CRA005817(https://ngdc.cncb.ac.cn/gsa/browse/CRA005817).

## Author Contributions

YY and MY conceived and designed the research. JZ and HZ acquired the data. JZ analyzed and interpreted the data. RL summed up the analysis. YY and JZ drafted the manuscript. YY, JZ, and SL revised the manuscript for important intellectual content. All authors have read the final manuscript.

## Conflict of Interest

MY was employed by Anhui Tongjisheng Biotechnology Company. The remaining authors declare that the research was conducted in the absence of any commercial or financial relationships that could be construed as a potential conflict of interest.

## Publisher’s Note

All claims expressed in this article are solely those of the authors and do not necessarily represent those of their affiliated organizations, or those of the publisher, the editors and the reviewers. Any product that may be evaluated in this article, or claim that may be made by its manufacturer, is not guaranteed or endorsed by the publisher.

## Footnotes

1
https://www.ncbi.nlm.nih.gov/genome/69090
