# Supplementary material for: Integration of Transcriptome and Metabolome Provides New Insights to Flavonoids Biosynthesis in Dendrobium huoshanense
Source: Front Plant Sci. 2022 Mar 14;13:850090. doi: 10.3389/fpls.2022.850090 (PMC8964182; doi:10.3389/fpls.2022.850090)
Supplement: Supplementary file 1 [file Table_1.DOCX]

**Table S1** **Genes IDs and primers used in the quantitative real-time PCR (qRT-PCR) experiments.**

| Primer_ID | Forward PCR Primer (5′-3′) | Reverse PCR Primer (5′-3′) |
| --- | --- | --- |
| *Actin* | TCCCAAGGCAAACAGAGAAA | GGCCACTAGCATATAGGGAAAG |
| LOC110101536 (*CHI*)  LOC110107833 (*CHS*)  LOC110103762 (*F3'5'H*1)  LOC110106061 (*F3'5'H*2)  LOC110095936 (*F3'H*1)  LOC110113268 (*F3'H*2)  LOC110115941 (*F3'H*3)  LOC110097028 (*FLS*1)  LOC110097219 (*FLS*2)  LOC110107557 (*FLS*3)  LOC110114894 (*FLS*4) | TGACCTCTTCTTTACGGCTC  CAGGATTACCAATAGCGAACACC  AAAGCCGGTTCTGCTACCAA  TCTCCGATGTTGTTCAATCCC  CGTCGCTTTCCAAATTCTTAGCA  TCACCAAACCTTACACGCCCTT  TGGCGATCCTCCCTCTCGT  CCGGCGAGCATTAATTATGG  ATTATTACCCTCCATGCCCTC  GGCTTATGCTAACTCTCCGACGA  GCGTCCAATCCCTTTCCGAT | TGATTTCTAACGGCACTCT  GTCGTTGCCAACCCGTCCTC  ACCAGCAACTTACGCACCA  TGGCCCACACATTTACCAG  AGCCATAAGCTCCATCACC  AAGAGTGAGCCCATAAGCCT  CCTCCTTCGCCTCAATCAGC  GCATGTAAATCTCACAAACTGAAA  TGGCATCAAACCAATGGTC  CACCGTTTCCCTCAGTGCAAC  CGATCTTGGCTCACTAGTTCCG |
