# Supplementary material for: Integration of Transcriptome and Metabolome Provides New Insights to Flavonoids Biosynthesis in Dendrobium huoshanense
Source: Front Plant Sci. 2022 Mar 14;13:850090. doi: 10.3389/fpls.2022.850090 (PMC8964182; doi:10.3389/fpls.2022.850090)
Supplement: Supplementary file 2 [file Data_Sheet_1.docx]

**Supplementary figures**


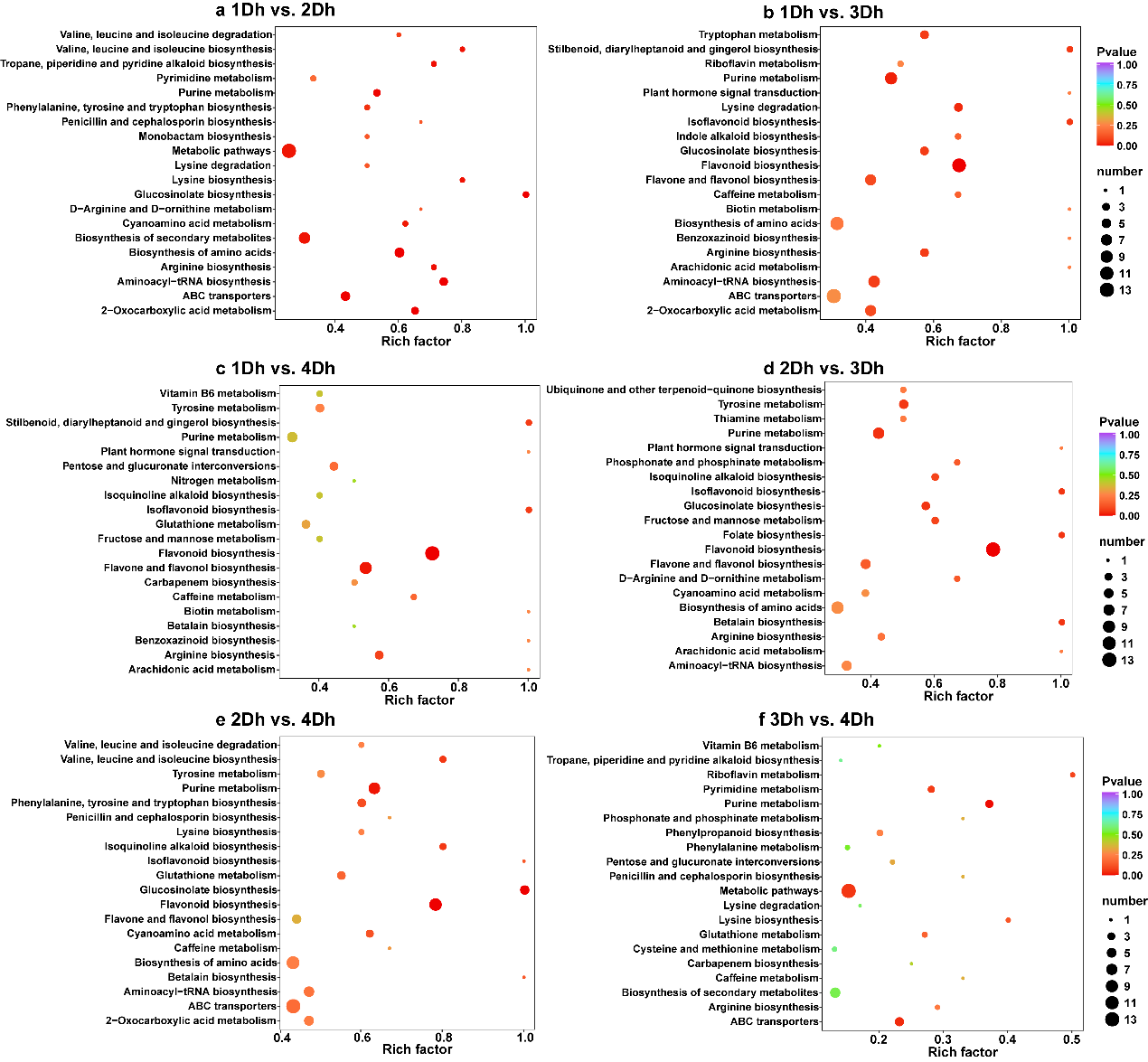


**Fig. S1 The KEGG pathway analysis of DAMs.** a 1Dh vs. 2Dh; b 1Dh vs. 3Dh; c 1Dh vs. 4Dh; d 2Dh vs. 3Dh; e 2Dh vs. 4Dh; f 3Dh vs. 4Dh.


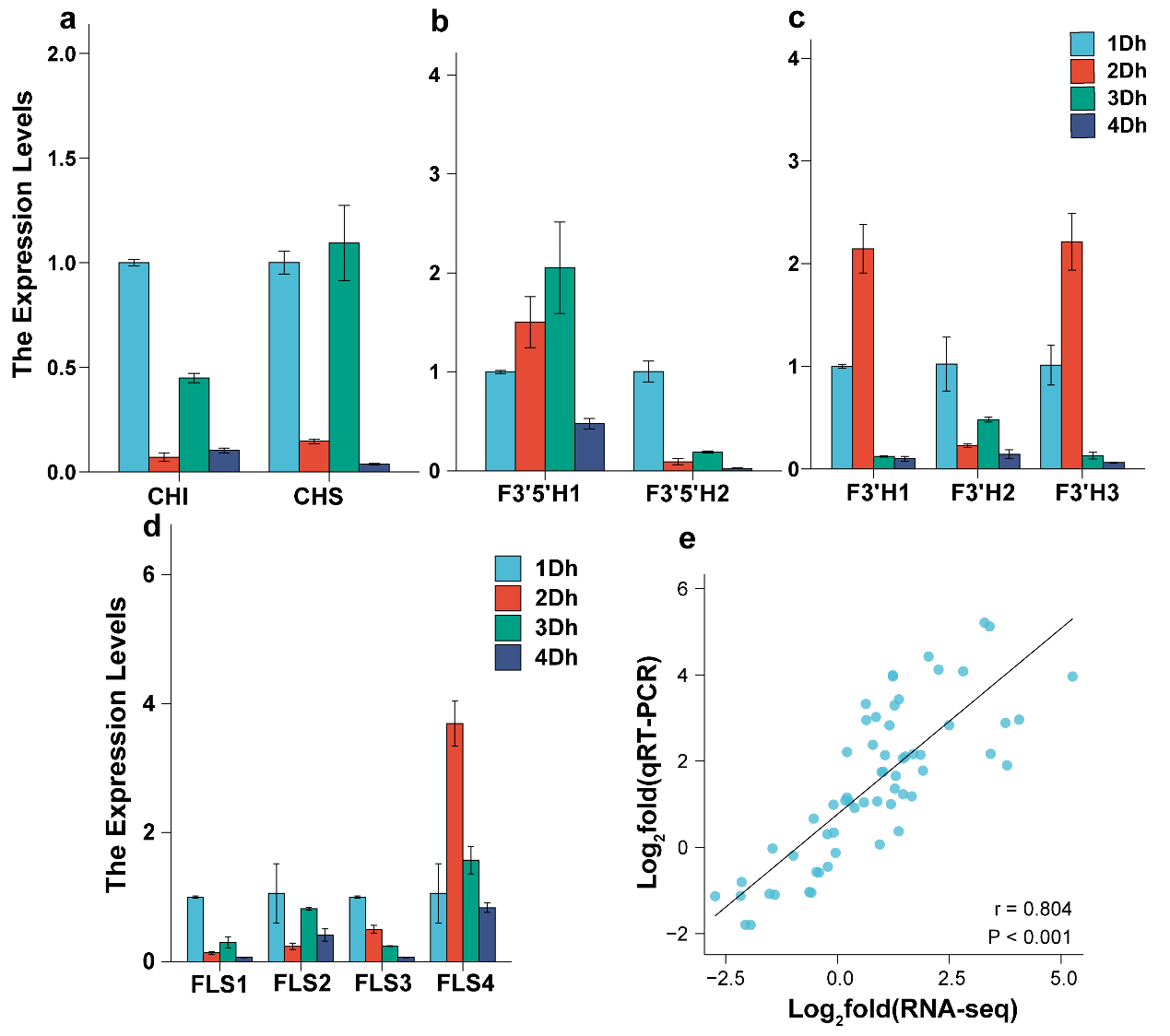


**Fig. S2 Analysis of the relative expression levels of 11 genes in flavonoid biosynthesis pathway by qRT-PCR.**
